# Mitigating climate-related health risks in Lebanon: implementation of national standards for climate-ready hospitals

**DOI:** 10.1093/intqhc/mzag150

**Published:** 2026-09-22

**Authors:** Elie Matta, Emile Whaibeh, Lea Kai, Jowy Abi Hanna, Alicia Abi Nader, Fadi Hindi, Riham Sanjakdar, Habib Abdo, Rouba El Khatib, Wael Al-Delaimy, Fayez Abdulla, Mazen Malkawi, Farah Jradi, Rana Amer, Joseph Zgheib, Myriam Mrad, Georges Abi Tayeh

**Affiliations:** Health and Environment Response Agency (HERA), Beirut, Lebanon; Doctoral School of Humanities, Letters, and Social Sciences, Lebanese University, Sin El Fil, Lebanon; Communication Skills Program, Faculty of Arts and Sciences, American University of Beirut, Beirut, Lebanon; Health and Environment Response Agency (HERA), Beirut, Lebanon; Public Health Department, Faculty of Health Sciences, University of Balamand, Beirut, Lebanon; Climate Change Office, United Nations Development Programme (UNDP), Ministry of Environment, Beirut Lebanon; Health and Environment Response Agency (HERA), Beirut, Lebanon; Public Health Department, Faculty of Health Sciences, University of Balamand, Beirut, Lebanon; Institut Supérieur de Santé Publique, Faculté de Médecine, Université Saint-Joseph, Beirut, Lebanon; Health and Environment Response Agency (HERA), Beirut, Lebanon; Public Health Department, Faculty of Health Sciences, University of Balamand, Beirut, Lebanon; Institut Supérieur de Santé Publique, Faculté de Médecine, Université Saint-Joseph, Beirut, Lebanon; Obstetrics and Reproduction Hôtel-Dieu de France University Hospital, Beirut, Lebanon; Obstetrics and Reproduction Hôtel-Dieu de France University Hospital, Beirut, Lebanon; Central Military Laboratories, Lebanese Army, Beirut, Lebanon; Agence Française de Développement (AFD), Beirut, Lebanon; School of Public Health, University of California San Diego, La Jolla, San Diego, CA, United States; Civil Engineering Department, Jordan University of Science and Technology, Irbid, Jordan; Ministry of Health and Prevention, United Arab Emirates; International Organization for Migration (IOM), Beirut, Lebanon; International Organization for Migration (IOM), Beirut, Lebanon; International Organization for Migration (IOM), Beirut, Lebanon; Health and Environment Response Agency (HERA), Beirut, Lebanon; Public Health Department, Faculty of Health Sciences, University of Balamand, Beirut, Lebanon; Health and Environment Response Agency (HERA), Beirut, Lebanon; Obstetrics and Reproduction Hôtel-Dieu de France University Hospital, Beirut, Lebanon

**Keywords:** climate change, healthcare standards, climate-resilient health systems, environmental sustainability, Lebanon, MENA region

## Abstract

**Background:**

Climate change increasingly disrupts healthcare systems worldwide by simultaneously increasing service demand and undermining infrastructure reliability. Hospitals face escalating risks from extreme weather, energy insecurity, and water scarcity, while contributing nearly 5% of global greenhouse gas emissions. These challenges are particularly acute in the Middle East and North Africa (MENA) region, where fragile health systems and resource constraints heighten vulnerability. Lebanon’s health sector exemplifies this intersection of climate risk and systemic fragility. This study aimed to develop nationally tailored standards for climate-resilient and environmentally sustainable healthcare facilities in Lebanon, translating international climate-health frameworks into actionable, context-specific guidance aligned with national legal and operational realities.

**Methods:**

A multiphase methodology was applied, including a scoping review of international standards and guidance (WHO, ISO 14001, ISO 9001, ISO 26000), structured consultations with key national and international stakeholders, and synthesis of draft standards into thematic areas. Clauses were aligned with existing Lebanese environmental and health legislation to ensure compliance and to prioritize urgent mandatory actions over longer-term strategic actions.

**Results:**

The resulting standards comprise 10 thematic areas covering governance, environmental management, climate emergency preparedness, community engagement, sustainable procurement, and performance monitoring. Legal screening enabled phased implementation, distinguishing urgent compliance-driven actions from longer-term strategic enhancements. Implementation assessment demonstrated that the standards could guide gap identification, cost estimation, and prioritization of climate-resilience investments at facility level.

**Conclusion:**

Climate-resilient healthcare standards for Lebanon provide a practical framework for strengthening service continuity during climate shocks while reducing the sector’s carbon footprint. Institutionalization and MoPH-led enforcement will be essential for long-term uptake.

## Introduction

Climate change is increasingly disrupting healthcare service delivery and infrastructure worldwide. Extreme weather events are putting hospitals at risk: a recent analysis found that one in 12 hospitals globally (∼16,000 facilities) faces high risk of total or partial shutdown from climate-related disasters if emissions continue unabated. The physical damage risk to hospitals from extreme weather has already risen by 41% since 1990 due to global warming [1].

Climate change is also increasing the strain on patient health, driving up demand for medical services. Globally, rising temperatures and extreme weather are already harming health: more than 5 million deaths each year are linked to abnormal hot and cold temperatures, and incidents like heatwaves, wildfires, and floods cause surges in injuries and illness [2]. Health systems must manage new climate-exacerbated disease burdens (such as heat stroke, respiratory issues from wildfire smoke, and expanding vector-borne diseases) even as disasters damage clinics and supply lines. The result, as the World Bank notes, is a double impact: climate change “significantly strains” health systems by simultaneously increasing demand for health services while impairing the system’s ability to respond [3]. In short, hospitals and clinics worldwide are facing more patients in crisis situations, often while coping with power outages, infrastructure damage, or staff and supply shortages due to climate shocks.

Ironically, while healthcare systems battle climate impacts, the sector itself is a major contributor to climate change through emissions and resource use. The healthcare sector accounts for nearly 5% of global greenhouse gas emissions [4]. These emissions stem from energy-intensive facilities, procurement, travel, and waste: for example, a large portion (∼71%) of healthcare’s climate footprint comes from its supply chain (production, transport, and disposal of medicines, devices, food, and equipment) rather than direct on-site emissions. Without changes, healthcare’s environmental footprint will grow: on our current trajectory, healthcare emissions could triple by 2050 [4], underscoring the need for greener, more efficient health systems even as we adapt to climate impacts.

### MENA region: vulnerabilities and health system challenges

The Middle East and North Africa (MENA) region is exceptionally vulnerable to climate change, with conditions that pose severe challenges to healthcare facilities and services. The region is warming at twice the global average rate, projected to see about +4°C by 2050 (vs ∼2°C globally) if high emissions continue [5]. This rapid heating, combined with intense humidity in coastal areas, means parts of MENA could experience extreme heat waves near or beyond human survivability by 2100. Heat exposure is already a critical issue and will dramatically worsen: heat-related mortality among older adults in MENA is expected to increase 8–20-fold by the late century compared to recent decades [6]. Beyond heat, water scarcity is an acute vulnerability: MENA has only around 1100 m³ of renewable water resources per person annually, versus ∼5000 m³ in Western Europe. This chronic water stress, alongside more frequent droughts and changing rainfall patterns, threatens sanitation and health service delivery (hospitals need reliable water for hygiene, dialysis, cooling, and others).

These vulnerabilities directly affect healthcare: extreme heat increases patient loads while threatening infrastructure; flooding damages hospitals and cuts access to care; water scarcity impairs operations; and many facilities lack backup power or robust construction.

### Lebanon: climate change challenges and healthcare

Lebanon exemplifies many of the climate-health challenges faced in the MENA region, compounded by the country’s economic struggles. Lebanon is highly vulnerable to climate disasters, ranking 44th out of 180 countries on a global climate risk index [6]. Economic crises have weakened the healthcare system, and heatwaves exacerbate chronic power outages, leading to frequent hospital failures. Climate change drives waterborne illnesses and threatens vector-borne disease resurgence, increasing service demand.

Lebanon’s healthcare system is pluralistic and highly privatized. The private sector accounts for 83% of the country’s total hospital-bed capacity, while the public hospital sector comprises 29 semi-autonomous hospitals [7]. Primary care is delivered through a national network coordinated and supported by the Ministry of Public Health (MoPH), mostly operated by nongovernmental organizations or municipalities [7]. The MoPH serves as the health sector’s steward and regulator and as an insurer of last resort for uninsured citizens, purchasing hospital care from contracted public and private providers [8]. The two sectors operate under distinct institutional frameworks: public hospitals are governed as semi-autonomous entities under Law No. 544/1996, whereas the establishment and licensing of private hospitals are regulated under Decree No. 9826/1962, as amended [8]. Despite these differences, both sectors are covered by the national hospital accreditation system, which was initiated in 2000 and is central to contracting arrangements and quality oversight for both sectors [9, 10]. Moreover, national environmental requirements governing areas such as healthcare-waste management and effluent discharge apply across healthcare facilities [11].

Within this regulatory context, Lebanon is taking steps to enhance the resilience of its healthcare sector and reduce its environmental footprint. One major focus has been securing reliable clean energy for health facilities in the face of electricity shortages. In recent projects, international organizations and the government have outfitted many health centers with solar power. Since 2023, UNICEF has equipped over 182 primary healthcare centers across Lebanon with solar energy systems (along with several regional health offices and a vaccine warehouse) [6]. United Nations Development Programme’s (UNDP’s) “Solar for Health” initiative is similarly installing solar photovoltaic hybrid systems in 10 public hospitals to provide critical power for operating rooms and wards [12]. This is an important initiative considering that healthcare emissions from diesel generators, which supplied the majority of Lebanon’s electricity in 2021–2022, account for ∼39% of electricity-related greenhouse gas emissions [13]. These measures act as both adaptation and mitigation: they improve resilience by keeping facilities running during heat waves or fuel crises, and they reduce carbon emissions and air pollution. However, these fragmented initiatives lack comprehensive system-level governance.

To address this gap, this study develops nationally tailored standards for climate-resilient and environmentally sustainable healthcare facilities in Lebanon. By adapting international frameworks to the country’s legal, economic, and infrastructural realities, the standards define clear operational requirements across governance, environmental management, emergency preparedness, and performance monitoring. The study further assesses the practical feasibility of implementing these standards through a hospital-level case study and proposes a phased approach aligned with national legislation. Together, these contributions offer a replicable model for translating global climate-health commitments into actionable healthcare practice in resource-constrained and crisis-affected settings.

## Materials and methods

The development of Lebanon’s national standards followed a structured, multi‑phase approach designed to translate international frameworks into practical guidance for Lebanese healthcare facilities.

The initial phase consisted of a structured review of various international documents. From this broader review, the study focused primarily on four core guidelines: ISO 14001 (environmental management), ISO 9001 (quality management), ISO 26000 (social responsibility), and the WHO guidance for climate-resilient and environmentally sustainable health facilities. Key clauses related to environmental protection, resource efficiency, climate readiness, social responsibility, and quality management were identified and synthesized using a thematic analysis approach, prioritizing relevance to healthcare operations, climate risk reduction, legal applicability, and feasibility in resource-constrained settings. Ten thematic areas were established (Table 1) and defined to align with core healthcare governance and operational functions. For each area, the team developed actionable clauses supplemented with activities and assessment tools to guide planning, implementation, and monitoring. Overlapping or redundant provisions were consolidated to enhance clarity and usability. The detailed clauses by theme can be found in Supplementary Table S1.

**Table 1:** Thematic areas, objectives, and example activities (total of clauses = 46).

Thematic area	Objectives and illustrative activities
Environmental and Sustainability Policy (4 clauses)	Establish a policy aligned with the facility’s mission that commits to environmental protection, pollution prevention, climate resilience, legal compliance, and continual improvement. Activities include drafting the policy with stakeholder input, appointing an environmental sustainability officer, and allocating resources for sustainability programmes.
Social Responsibility (3 clauses)	Uphold ethical obligations to patients, staff, communities, and the environment. Activities include ensuring equitable access to care, fair labor practices, community engagement, transparency, and inclusion in decision‑making.
Air Quality Management (5 clauses)	Monitor and control indoor and outdoor air pollutants, implement ventilation and filtration systems, and minimize emissions from generators and incinerators.
Water and Sanitation Management (6 clauses)	Ensure safe, efficient use of water through conservation, recycling and rainwater harvesting; maintain sanitation and infection control; plan for water scarcity.
Waste Management (6 clauses)	Implement segregation, safe handling and disposal of general, hazardous and biomedical waste; promote recycling and reduction at source; track waste quantities.
Energy Conservation and Efficiency (5 clauses)	Increase energy efficiency through audits, insulation, efficient equipment and lighting; integrate renewable energy (e.g., solar PV systems). Solarizing health facilities can improve resilience and cut emissions.
Climate Emergency Preparedness and Resource Planning (6 clauses)	Conduct risk assessments for heat waves, floods, wildfires, and supply disruptions; develop emergency response plans; secure backup power and water; integrate climate scenarios into facility design.
Community Engagement, Risk Communication and Capacity‑Building (4 clauses)	Engage communities in climate‑health planning, communicate risks and adaptation measures, and train staff on climate‑sensitive care. The IOM/MoPH needs assessment highlighted the need for training and updated curricula.
Sustainable Procurement and Supply Chain Responsibility (2 clauses)	Adopt procurement policies favoring low‑carbon, responsibly sourced products; engage suppliers on sustainability; monitor supply‑chain emissions.
Environmental Performance Monitoring and Continuous Improvement (5 clauses)	Establish indicators to track energy use, emissions, water consumption, waste generation and progress on sustainability goals; regularly audit performance and review targets; implement corrective actions.

The draft framework was then reviewed in one structured consultation round involving 15 representatives from various local and regional partners: the WHO Regional Office for the Eastern Mediterranean, UNDP, the Ministry of Environment, the MoPH, the International Organization for Migration, Agence Française de Développement, and regional and international university partners, together with representatives from the private hospital participating in the feasibility assessment. Participants were selected purposively to capture regulatory, technical, development-partner, and facility-level implementation perspectives.

The draft standards were circulated alongside a structured feedback matrix that allowed local experts and international partners to provide detailed comments and indicate relative priority levels. The matrix asked participants to rate the urgency of each clause as short-term (immediate to 1 year), medium-term (1–3 years), or long-term (>3 years), and to provide comments on the proposed timeframe and implementation considerations within their areas of expertise. Written and verbal feedback was consolidated and used to refine the wording, feasibility, and prioritization of the draft standards. This consultation was advisory rather than a formal consensus process.

### Practical implementation of the national standards

To assess the operational feasibility of the drafted standards, a facility-level implementation assessment was conducted by the Health and Environment Research Agency, in collaboration with Hôtel-Dieu de France—a private hospital in Beirut, Lebanon. The study reviewed all 10 thematic areas of the standards but focused specifically on five operational domains: Air Quality Management, Water and Sanitation Management, Waste Management, Energy Conservation, and Environment and Climate Emergency Preparedness. This process enabled systematic identification of compliance gaps, estimation of implementation costs, and prioritization of actions based on urgency, feasibility, and regulatory requirements.

Based on legal cross-referencing (or legal mapping) and implementation considerations, clauses were categorized into two implementation phases:

- Phase 1 prioritizes urgent clauses mandated by Lebanese environmental and health legislation (e.g. biomedical waste segregation, potable water safety, air quality thresholds);
- Phase 2 encompasses longer-term measures such as solar energy integration, greywater recycling, and systematic supply-chain decarbonization.

This approach ensures legal compliance while gradually building climate resilience and environmental sustainability capacity across facilities.

To determine which standards must be prioritized for immediate implementation, a legal screening process was conducted to map each clause against existing Lebanese environmental and health legislation. Clauses directly required under national laws and decrees were classified as Phase 1 (Urgent and Mandatory), while nonbinding but strategically important measures were classified as Phase 2 (Strategic Enhancements). Clauses were cross-referenced with Lebanese environmental and health laws, including Law 444/2002 on Environmental Protection, Law 78/2018 on Air Quality, Law 80/2018 on Integrated Solid Waste Management, Water Law 221/2000, Decree-Law 13389/2004 on medical waste classification, the EIA Decree 8633/2012, the SEA Decree 8213/2012, and the Environmental Compliance for Establishments Decree 8471/2012, among others. Together, these steps ensured that the standards were evidence-based, context-specific, legally grounded, and practically implementable within the Lebanese healthcare system.

## Results

The results of the study are presented in three parts: (1) an overview of the thematic structure of the standards, (2) legal prioritization of clauses into urgent and strategic phases, and (3) findings from the facility-level implementation assessment. These results illustrate how the standards translate international guidance into legally grounded and operationally actionable requirements.

### Thematic structure of the standards

The final standards comprised 10 thematic areas and 46 clauses. The domains with the largest number of clauses were *Water and Sanitation Management*, *Waste Management*, and *Climate Emergency Preparedness and Resource Planning* (6 clauses each), followed by *Air Quality Management*, *Energy Conservation and Efficiency*, and *Environmental Performance Monitoring and Continuous Improvement* (5 clauses each). This distribution reflects the emphasis placed on operational continuity, environmental compliance, and emergency readiness.

### Legal mapping/screening

Legal screening revealed a clear distinction between clauses that are already mandated under Lebanese environmental and health legislation and those that represent best-practice enhancements beyond minimum compliance. Supplementary Table S2 summarizes how each thematic area was divided into urgent, legally required actions (Phase 1) versus strategic, nonbinding actions (Phase 2).

Phase 1 actions predominantly relate to pollution control, waste management, potable water safety, air quality compliance, and environmental monitoring, which are areas where legal obligations already exist, but implementation remains inconsistent across facilities. On the other hand, phase 2 actions largely focus on structural and systemic improvements, including renewable energy integration, sustainable procurement, community engagement, and long-term emergency preparedness, which require greater financial investment and institutional capacity. To illustrate the financial implications of prioritizing urgent actions, Fig. 1 presents the estimated Phase 1 budget distribution for a private hospital in Mount Lebanon with ∼400 patient beds. It is worth noting that these figures, drawn from estimates presented by two representatives from HDF, are preliminary in nature. Comprehensive cost analyses and operational feasibility assessments are currently underway to formally validate and expand upon these initial financial projections.

**Figure 1:**
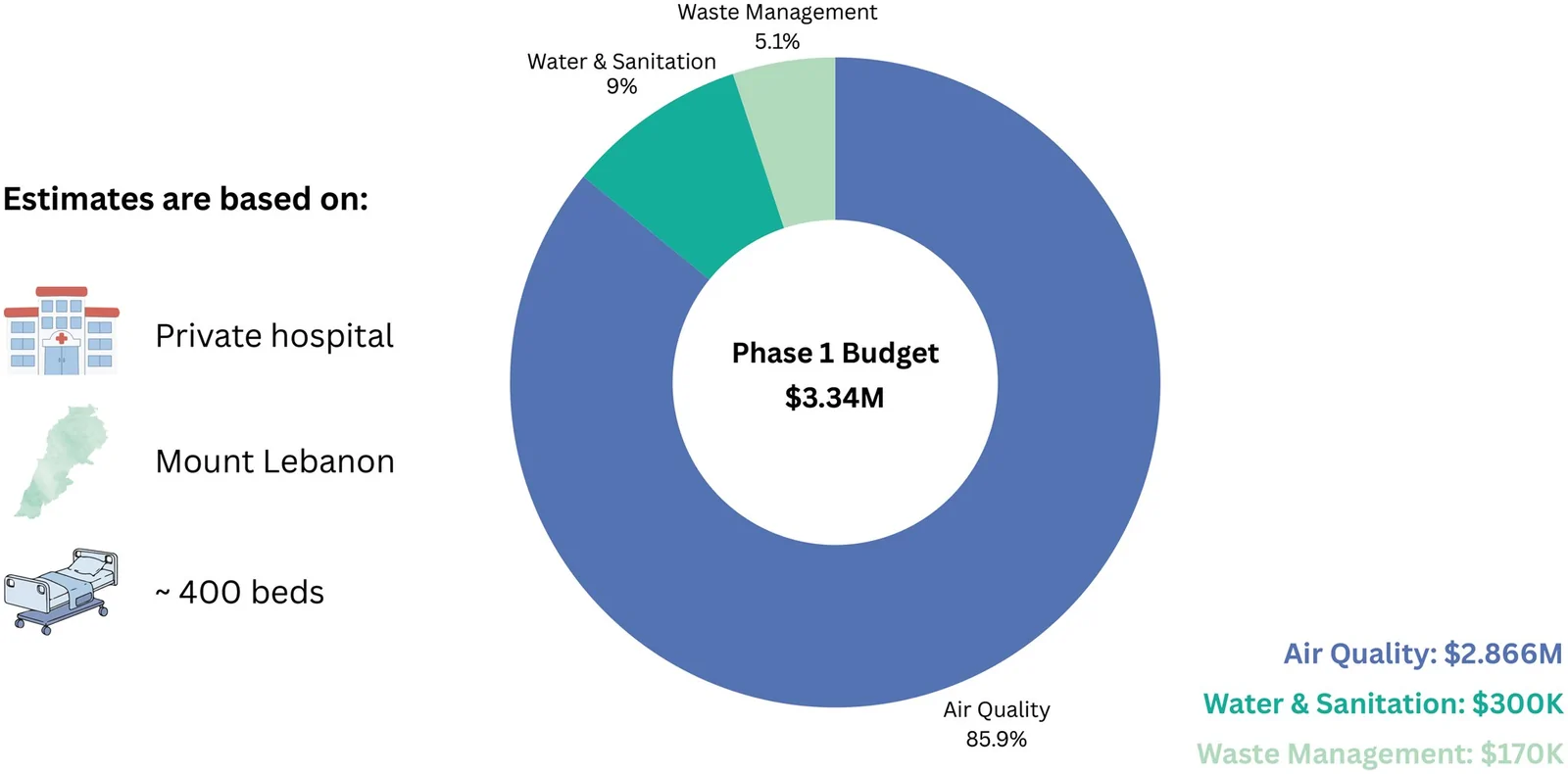
Distribution of Phase 1 implementation costs across thematic areas for a private hospital in Mount Lebanon with ∼400 patient beds.

The distribution highlights that air quality management represents the largest share of Phase 1 costs, reflecting regulatory requirements related to emissions control, ventilation, and monitoring, while water, sanitation, and waste management account for smaller but essential compliance-driven investments.

### Hospital-based implementation assessment

In the pilot assessment at HDF, the standards were used to identify implementation gaps across five operational domains: air quality, water and sanitation, waste management, energy, and climate emergency preparedness. The most frequent gaps were related to documentation, monitoring systems, and emergency preparedness planning, rather than the complete absence of basic infrastructure. Phase 1 costing showed that air quality management accounted for the largest share of urgent implementation costs, followed by water/sanitation and waste-related actions.

## Discussion

### Statement of principal findings

The main contribution of this study is not simply that it adapts global guidance, but that it converts broad climate-health principles into a phased national standard linked to Lebanese legal obligations. This is particularly important in settings where facilities face severe resource constraints and cannot implement all climate-related actions at once. By systematically cross-referencing each clause against existing Lebanese environmental and health legislation, the standards introduce a clear prioritization framework that reduces ambiguity for healthcare facilities, supports regulatory compliance, and enables the MoPH to align climate-health objectives with existing licensing and accreditation mechanisms [14]. While evidence regarding the broader impact of hospital accreditation on healthcare quality remains context-dependent [15], embedding clear environmental priorities into these regulatory structures positions sustainability as a fundamental dimension of healthcare quality [16].

This contextualization represents a key distinction from existing international instruments. WHO guidance provides a global framework and a set of suggested interventions for climate-resilient and environmentally sustainable healthcare facilities, while explicitly recognizing that these must be adapted to local realities and requirements [17]. The *Global Green and Healthy Hospitals (GGHH) Agenda* provides a goal-based framework comprising 10 interconnected areas of action for hospitals and systems worldwide [18], whereas ISO 14001 specifies sector-neutral requirements for establishing and maintaining an environmental management system applicable to organizations of any type or sector [19]. Although these instruments support local adaptation and prioritization, they do not map individual actions to Lebanese legal obligations, distinguish legally mandated actions from strategic enhancements under Lebanese law, or provide Lebanon-specific facility-level cost estimates. The proposed framework addresses these gaps by mapping each clause to national legislation, classifying actions according to legal priority, defining verification criteria, and assessing implementation feasibility through facility-level costing.

A notable finding from the pilot assessment was that the main implementation gaps were not primarily infrastructural, but managerial. Deficiencies were concentrated in documentation, monitoring, and preparedness planning. These findings are consistent with implementation science evidence indicating that adoption of quality interventions depends on organizational capacity, leadership, stakeholder engagement, and the local implementation context [20]. They also suggest that early progress may be achieved through governance reforms, audit tools, and standard operating procedures before major capital investments are required. In fragile systems such as Lebanon’s, this is operationally important because it identifies lower-cost entry points for implementation. The Lebanese standards advance this agenda by providing actionable clauses and verification criteria that go beyond the high-level goals of frameworks such as the *GGHH Agenda* [21], guiding facilities step-by-step rather than leaving them to interpret broad aspirational targets.

Energy conservation and renewable energy are particularly relevant given Lebanon’s chronic electricity shortages. Solar programs demonstrate that combining renewable energy with efficiency improves reliability and reduces emissions. The standards align with global decarbonization agendas—supply-chain emissions comprise ∼70% of healthcare’s footprint—and Lebanon’s inclusion of sustainable procurement directly addresses this challenge. Beyond technical measures, climate topics remain largely absent from clinical training; the standards’ emphasis on capacity-building and risk communication aims to mainstream climate considerations throughout healthcare education and practice.

### Interpretation within the context of the wider literature

Hospitals implementing climate-smart frameworks report measurable resilience and cost benefits. Boston Medical Center reduced energy use by 35% and carbon emissions by 91%, saving ∼$40 million annually [22]. Hospital Sírio-Libanês in Brazil achieved 40% energy reduction and 15% emissions drop [23]; a South African clinic’s rainwater harvesting improved water security [24]. However, fragile healthcare contexts often lack stable financing, continuous electricity, and strong regulatory enforcement. The phased approach prioritizes legal compliance, low-cost operational measures, and governance processes, enabling facilities to focus first on mandatory actions before pursuing structural investments. Hospitals in 17 countries implementing climate-smart measures reported lower emissions alongside improved patient safety, collectively saving millions through energy and waste reductions [25]. Reducing air pollution and ensuring reliable power and water during climate shocks ensures care continuity when patients need it most. Viewed through a healthcare-quality lens, these interventions can be linked to measurable operational indicators, including continuity of electricity and water supply, energy consumption, compliance with air- and water-quality requirements, waste-management performance, and implementation costs. Their expected quality benefits include safer care, fewer climate-related service disruptions, and improved operational efficiency [24, 26].

Low- and middle-income countries often face resource constraints, legal and regulatory gaps, and political and economic instabilities that can weaken health-system governance and limit their capacity to address climate-related hazards. Although national contexts may vary substantially, the principal transferable contribution of this study lies in the structured method for developing locally grounded standards. The process comprises five replicable steps: (1) selecting relevant international frameworks; (2) mapping individual requirements to national legislation and regulatory responsibilities; (3) distinguishing legally mandated actions from strategic enhancements; (4) translating requirements into auditable clauses and verification criteria; and (5) assessing the feasibility and costs at the facility level. Further piloting across facilities of different sizes, ownership types, and levels of care would be needed before nationwide adoption.

### Strengths and limitations

Implementation capacity varies substantially across Lebanese healthcare facilities due to financial resources, technical expertise, and workforce availability. Baseline facility-level data on energy consumption may be incomplete, hindering performance monitoring; proposed monitoring clauses are designed to address data gaps. Health facilities vary widely in size and governance, requiring customization based on facility type. Adoption is currently voluntary; however, the legal mapping and phased approach provide a pathway for institutionalization through existing regulatory instruments. Lebanon’s ongoing economic crisis, recurrent governmental paralysis, regional conflict, and security instability constitute additional contextual constraints. These conditions may restrict stakeholder engagement, delay regulatory adoption and enforcement, disrupt implementation, and alter the cost assumptions underlying the implementation assessment. In addition, the pilot was conducted in a single, large private hospital, which limits the generalizability of the cost findings to public and smaller facilities. As climate risks and technologies evolve, periodic revision will be necessary.

### Implications for policy, practice, and research

Climate change threatens healthcare systems in Lebanon and across MENA, particularly in contexts marked by economic strain, infrastructure fragility, and governance challenges. The standards translate global climate-health principles into actionable, legally grounded guidance. By embedding climate adaptation and sustainability within healthcare governance, the standards reposition resilience as a core component of quality healthcare delivery. The findings support phased implementation, institutionalization, and scale-up of the standards. For the Lebanese context, recommendations for the future phases of the project are outlined in Table 2.

**Table 2:** Recommendations for future phases in Lebanon.

Conduct feasibility assessments Undertake feasibility studies with hospitals to identify the financial requirements and technical needs for implementing the necessary changes or installations required under the standards. This will help prioritize investments, plan phased implementation, and align funding sources.
Institutionalize the standards The MoPH shall incorporate the standards into licensing, accreditation, and funding requirements. Establishing a national oversight committee can monitor implementation and provide technical support.
Build human capacity Under MoPH coordination, integrate climate‑health topics into medical, nursing, and public‑health curricula. Provide continuing professional development for existing staff on emergency preparedness, sustainable procurement, and environmental management.
Develop data systems With the help of the MoPH, establish national reporting mechanisms for energy use, emissions, water consumption, and waste. Digital dashboards can help facilities benchmark progress and identify priority interventions.
Engage communities and suppliers Community engagement is essential for risk communication and trust‑building. Facilities shall also work with suppliers to adopt low‑carbon procurement practices, thereby addressing the substantial supply‑chain emissions.
Periodic review and learning Climate science and technologies evolve quickly. The standards shall be revisited every 3–5 years to incorporate new evidence, adjust targets, and share lessons learned. Peer‑learning networks among Lebanese health facilities can accelerate innovation.

## Conclusions

The Lebanese standards framework provides a practical, context-specific pathway for building climate-resilient and environmentally sustainable healthcare systems. By linking international climate-health guidance to national legal obligations and establishing a phased implementation approach, the standards reduce implementation ambiguity and enable healthcare facilities to systematize climate actions. The integration of governance reforms, low-cost operational measures, and strategic infrastructure investments creates a realistic pathway for resource-constrained facilities to advance climate resilience without requiring immediate large-scale investment. Formal institutionalization through national regulatory mechanisms, sustained technical and financial support, and iterative revision as climate risks and technologies evolve are essential for long-term success. The approach offers a transferable model for other low- and middle-income countries facing similar challenges, demonstrating that rigorously grounded, locally adapted standards can enable healthcare systems to address climate risks while advancing environmental and public health co-benefits.

## Supplementary Material

mzag150_Supplementary_Data

## Data Availability

The data underlying this article are available from the corresponding author upon reasonable request. Some facility-level and stakeholder consultation data are not publicly available because they contain confidential institutional information.

## References

[1] Ramos M , Adebayo B. Thousands of hospitals at high risk from climate change, warns report. *Context*. 2023, December 2. https://www.context.news/climate-risks/thousands-of-hospitals-at-high-risk-from-climate-change (date last accessed, 29 September 2026)

[2] Gaden M Tackling climate change: Health care professionals can do more by using less. *The Commonwealth Fund*. 2024. 10.26099/G5FG-N380 1

[3] The Health Policy Partnership. The nexus between climate change and healthcare. *The Health Policy Partnership*. n.d. https://www.healthpolicypartnership.com/the-nexus-between-climate-change-and-healthcare/

[4] Othering & Belonging Institute. Lebanon case study| Climate refugees. *Othering & Belonging Institute*. 2023. https://belonging.berkeley.edu/climatedisplacement/case-studies/lebanon (date last accessed, 29 September 2026)

[5] Red Cross Red Crescent Climate Centre. Country profile: Lebanon [PDF]. *Red Cross Red Crescent Climate Centre*. 2024. https://www.climatecentre.org/wp-content/uploads/RCCC-Country-profiles-Lebanon_2024_final.pdf (date last accessed, 29 September 2026)

[6] UNICEF Lebanon. Solar energy for health: powering primary healthcare centres in Lebanon. *UNICEF*. 2023. https://www.unicef.org/lebanon/solar-energy-health (date last accessed, September 29 2026)

[7] Ministry of Public Health, Lebanon. Lebanon health sector strategy—Vision 2030: Annex 2, Health sector overview. *Ministry of Public Health*. 2023. https://www.moph.gov.lb/userfiles/files/About%20MOPH/StrategicPlans/National-Health-Strategy%E2%80%93Vision2030/Lebanon-Health-Sector-Strategy_Annex2.pdf (date last accessed, September 29 2026)

[8] World Health Organization Regional Office for the Eastern Mediterranean. Understanding the Private Health Sector in Lebanon. Cairo: WHO Regional Office for the Eastern Mediterranean; 2024. https://applications.emro.who.int/docs/9789292742225-eng.pdf

[9] Mansour W , Boyd A, Walshe K. The development of hospital accreditation in low- and middle-income countries: a literature review. Health Policy Plan 2020;35:684–700. 10.1093/heapol/czaa011 PMC7294243 32268354

[10] Ammar W , Wakim IR, Hajj I. Accreditation of hospitals in Lebanon: a challenging experience. East Mediterr Health J 2007;13:138–49. 17546916

[11] Ministry of Public Health, Lebanon. Lebanon Health Resilience Project: Environmental and Social Management Framework. *Beirut: Ministry of Public Health*; 2019. https://www.moph.gov.lb/userfiles/files/Programs%26Projects/LHRP%20-%20ESMF.pdf

[12] United Nations Development Programme. Solar for health strategy report [PDF]. *UNDP*. 2022, November. https://www.undp.org/sites/g/files/zskgke326/files/2022-11/SOLAR%20FOR%20HEALTH%20STRATEGY%20REPORT.pdf (date last accessed, 29 September 2026)

[13] The Commonwealth Fund. Greening health care’s safety net: case study [Case study]. *The Commonwealth Fund*. 2023, August. https://www.commonwealthfund.org/publications/case-study/2023/aug/greening-health-care-safety-net (date last accessed, 29 September 2026)

[14] Global Environment Facility. Rays of hope: greening the reconstruction of Lebanon. *Global Environment Facility*. n.d. https://www.thegef.org/news/rays-hope-greening-reconstruction-lebanon (date last accessed, 29 September 2026)

[15] Lewis K , Hinchcliff R. Hospital accreditation: an umbrella review. Int J Qual Health Care 2023;35:mzad007. 10.1093/intqhc/mzad007 PMC9950788 36738157

[16] Ossebaard HC , Lachman P. Climate change, environmental sustainability and health care quality. Int J Qual Health Care 2021;33:mzaa036. 10.1093/intqhc/mzaa036 PMC7197527 33693895

[17] World Health Organization. WHO Guidance for Climate-Resilient and Environmentally Sustainable Health Care Facilities. Geneva: World Health Organization. https://www.who.int/publications/i/item/9789240012226

[18] Karliner J , Guenther R. Global *Green and Healthy Hospitals agenda: A comprehensive environmental health agenda for hospitals and health systems around the world*. Health Care Without Harm. 2011.

[19] International Organization for Standardization. *ISO 14001:2015: Environmental Management Systems—Requirements With Guidance for Use*. Geneva: ISO, 2015. https://www.iso.org/standard/60857.html

[20] Braithwaite J , Marks D, Taylor N. Harnessing implementation science to improve care quality and patient safety: a systematic review of targeted literature. Int J Qual Health Care 2014;26:321–9. 10.1093/intqhc/mzu047 24796491

[21] Prabhakaran P , Armstrong F, Karliner J. Decarbonising the healthcare sector: a roadmap for G20 countries [T20 Policy Brief]. *Think20 India*. 2023, June. https://t20ind.org/research/decarbonising-the-healthcare-sector/

[22] Joshi KJ , Sharma L, Singh J et al Advancing solar energy for primary healthcare in developing nations: addressing current challenges and enabling progress through UNICEF and collaborative partnerships. Cureus 2024;16:e51571. 10.7759/cureus.51571 PMC10835743 38313940

[23] Health Care Without Harm. *Health care climate learning initiative: Case studies from around the globe*. 2024. https://global.noharm.org/resources/climate

[24] World Bank. Climate-Smart Health Care: Low-Carbon and Resilience Strategies for the Health Sector. World Bank Group, 2017. https://documents.worldbank.org/en/publication/documents-reports/documentdetail/871991517932929570

[25] Global Green and Healthy Hospitals. *Global Green and Healthy Hospitals network highlights 2024*. 2025. https://greenhospitals.org/sites/default/files/2025-03/Global%20Green%20and%20Healthy%20Hospitals%20network%20highlights%202024.pdf

[26] Global Green and Healthy Hospitals. *38 case studies, 17 countries, 1 goal: Decarbonize health care*. 2024. https://greenhospitals.org/news/38-case-studies-17-countries-1-goal-decarbonize-health-care

