# Supplementary material for: Mitigating climate-related health risks in Lebanon: implementation of national standards for climate-ready hospitals
Source: Int J Qual Health Care. 2026 Sep 22;38(4):mzag150. doi: 10.1093/intqhc/mzag150 (PMC13648154; doi:10.1093/intqhc/mzag150)
Supplement: Supplementary file 1 [file mzag150_supplementary_data.docx]

**Supplementary Tables**

**Supplementary Table 1 – Detailed Clauses by Theme**

| **Theme** | **Clauses** |
| --- | --- |
| **Environmental & Sustainability Policy** | **1.1**  The healthcare facility shall establish, implement, and continually improve an Environmental and Sustainability Policy that is proportional to the facility’s purpose and context, reflects the scale and environmental impacts of its activities, and provides a clear framework for setting and reviewing objectives.  The facility’s Environmental and Sustainability Policy shall commit the facility to: (1) protecting the environment and preventing pollution, (2) strengthening emergency preparedness and climate resilience by anticipating, preparing for, and mitigating the impacts of climate change on the facility and served population, (3) complying with all legal requirements and other obligations, and (4) driving ongoing improvement in environmental and climate performance through systematic documentation, monitoring, and reporting. |
|  | **1.2**  The healthcare facility’s top management shall demonstrate commitment and accountability for environmental and climate resilience and patient-safety outcomes by ensuring appropriate resources are allocated, communicating objectives, managing associated risks, engaging personnel and partners, and driving continual improvement. |
|  | **1.3**  The healthcare facility shall determine the external and internal issues that shape its Environmental and Sustainability Policy. External issues include environmental and climate conditions that could disrupt operations and service capacity, while internal issues include the facility’s activities, services, and greenhouse gas emissions that may harm the surrounding environment. This two-way assessment shall inform the policy’s scope, objectives, and actions. |
|  | **1.4**  The healthcare facility shall identify and actively engage and collaborate with interested parties affected by or able to influence the Environmental and Sustainability Policy and consider their needs, expectations, roles, and capacities in supporting the facility’s climate preparedness, mitigation, and other activities. |
| **Social Responsibility** | **2.1**  The healthcare facility shall integrate environmental and climate responsibility into its governance by remaining informed on the historical evolution and current trajectories of climate change, including the healthcare sector’s share of greenhouse gas emissions, allocating the resources needed to mitigate its impact, preparing for climate-related sequelae, and promoting sustainable development, thereby fulfilling its ethical and societal duty to safeguard the community it serves. |
|  | **2.2**  The healthcare facility shall comply with all environmental laws, regulations, and voluntary commitments, and uphold ethical conduct in any activity that affects environmental performance or climate adaptation. |
|  | **2.3**  The healthcare facility shall establish metrics for environmental impact and climate-resilience objectives, monitor and document performance, disclose relevant results to interested parties, and conduct regular reviews to drive continual improvements. |
| **Air Quality Management** | **3.1**  The healthcare facility shall identify and quantify on-site air emission sources. |
|  | **3.2**  The healthcare facility shall apply best-available controls or cleaner fuels to keep particulate matter, nitrogen oxides, volatile organic compounds (VOC), and other pollutants within the national ELVs. |
|  | **3.3**  The healthcare facility’s ventilation systems shall include appropriate filtration and real-time indoor air quality monitoring. |
|  | **3.4**  Using recognized methods, the healthcare facility shall, every year, quantify greenhouse gas emissions from (1) on-site fuel and fleet, (2) purchased energy, (3) supply-chain, transport, and waste |
|  | **3.5**  The healthcare facility shall set air quality targets, disclose progress continuously, and implement transport and procurement strategies that reduce GHG emissions. |
| **Water and Sanitation Management** | **4.1**  The healthcare facility shall routinely inspect and maintain all elements of its water and wastewater systems, including pipes, valves, pumps, storage tanks, and back-flow devices. |
|  | **4.2**  The healthcare facility shall develop a climate resilient water safety plan that ensures potable-water safety. |
|  | **4.3**  The healthcare facility shall manage stormwater run-off and monitor sewer systems to prevent overflow and contamination. |
|  | **4.4**  The healthcare facility shall ensure wastewater is safely managed through on-site treatment (e.g., septic tank followed by drainage pit) or discharged through an authorized sewerage or treatment system in compliance with applicable environmental regulations. |
|  | **4.5**  The healthcare facility shall develop and communicate a water-conservation policy, reinforce it with clear signage for patients, visitors and staff, and embed conservation measures and awareness into staff orientation and ongoing personnel training. |
|  | **4.6**  When rainfall or greywater recovery is feasible, the healthcare facility may install and routinely inspect covered rainwater or greywater systems, limit their use to non-potable purposes (i.e., toilet flushing, outdoor cleaning, irrigation), and ensure wastewater disposal practices prevent any cross-contamination of local drinking-water sources. |
| **Waste Management** | **5.1**  The healthcare facility shall periodically assess the types, volumes, and hazards of all waste it generates and maintain a written plan that sets reduction, recycling, treatment, and disposal pathways for each category. |
|  | **5.2**  The healthcare facility shall establish a segregated collection of different types of waste according to hazards, using clearly labelled, color-coded containers for infectious, sharps, chemical, pharmaceutical, recyclable, and non-hazardous waste. |
|  | **5.3**  On-site incineration shall be phased out in favor of non-burn techniques (e.g. autoclaving for infectious waste). Residual cytotoxic, pharmaceutical or chemical waste shall be transferred only to licensed facilities (domestic or foreign) that meet higher treatment standards. |
|  | **5.4**  The healthcare facility shall implement and monitor a waste reduction programme that embeds good practices through ongoing waste management training for all staff. |
|  | **5.5**  The healthcare facility shall minimize single-use plastic waste by adopting reusable and refillable alternatives, incorporating low-plastic criteria into procurement and catering contracts, and tracking progress as part of the facility’s waste reduction program. |
|  | **5.6**  The healthcare facility shall evaluate how climate-related hazards could disrupt waste storage, handling, and disposal and build contingency measures to ensure every waste stream is safely managed when normal conditions are compromised. |
| **Energy Conservation and Efficiency** | **6.1**  The healthcare facility shall maintain a documented energy-management plan that measures baseline consumption, sets departmental reduction targets, tracks performance, and is reviewed periodically for continual improvement. |
|  | **6.2**  The facility shall optimize on-site alternative sources of energy (e.g.: solar photovoltaics (PV)), switch from diesel generators to lower-carbon fuels when feasible. |
|  | **6.3**  The healthcare facility shall adopt energy efficiency measures such as maximizing daylighting, installing high-efficiency LED fixtures, and selecting energy-efficient appliances and refrigeration units. |
|  | **6.4**  The health facility’s building shall be thermally insulated, and where structurally feasible, incorporate passive-design features such as high ceilings, operable windows, or skylights to maximize natural ventilation. |
|  | **6.5**  The facility shall nurture an energy-saving culture by providing induction and refresher training for personnel, clear signage, and incentive schemes to prompt staff members to switch off lights, computers, and equipment and to unplug devices when not in use. |
| **Environment and Climate Emergency Preparedness and Resource Planning** | **7.1**  The healthcare facility shall identify, across the full life cycle of its activities and services, the environmental impact it controls or can influence, apply objective criteria to prioritize those with the greatest significance, and plan and allocate resources to mitigate the associated risks. |
|  | **7.2**  To safeguard operations during climate-related emergencies, the healthcare facility shall assess its energy profile, reducing demand where feasible and reinforcing supply where it is vulnerable. It shall prioritize the lower-cost, highest-impact energy sources and develop and maintain a risk-management plan for intermittent energy disruptions or system failures |
|  | **7.3**  The healthcare facility shall embed, within its climate-resilient water safety plan, long-term drought management, identify and secure alternative water sources, set measures to ensure uninterrupted and safe supply, and provide real-time monitoring of water quantity with pre-defined rationing protocols to sustain critical functions until normal service is restored. |
|  | **7.4**  The healthcare facility shall develop an anticipatory disaster and climate-resilience plan that identifies climate and environmental hazards and vulnerable zones, schedules for preparing infrastructure in advance of flood seasons to avoid overflows, protects equipment and supplies, secures and supports HVAC ductwork to prevent failure, and sets clear evacuation and post-flood protocols. |
|  | **7.5**  Prior to the extreme weather hazards, the healthcare facility shall maintain and routinely rotate a secure stockpile of essential pharmaceuticals, medical consumables, and critical supplies to sustain core services throughout climate-related or other emergencies. |
|  | **7.6**  The healthcare facility, during the recovery stage, shall dry, clean, and remediate affected areas without delay to prevent dampness-related problems such as mold growth, which can compromise indoor air quality. |
| **Community Engagement, Risk Communication, and Capacity Building** | **8.1**  The healthcare facility shall define its internal and external communication requirements for environmental and climate-health matters, assign responsibilities, and ensure timely distribution of relevant information to staff, management, regulators, patients, and community partners. |
|  | **8.2**  The healthcare facility shall maintain open, two-way communication with patients, personnel, suppliers, community representatives, and authorities, sharing timely information on risks and safety measures and using stakeholder feedback to enhance service quality, patient safety, environmental performance, and community-health initiatives. |
|  | **8.3**  The healthcare facility shall partner with local communities to deliver and support public health actions (e.g.: vaccination drives, screening campaigns, health-education sessions), that empower residents to make informed decisions on prevention, treatment, wellness, and the management of climate-related health risks. |
|  | **8.4**  The healthcare facility shall support professional development for all personnel by offering ongoing training, skill-building, and career-growth opportunities that include climate-resilient healthcare practices and emergency preparedness. |
| **Sustainable Procurement and Supply Chain Responsibility** | **9.1**  The healthcare facility shall apply environmental criteria to every purchasing decision, favoring suppliers that uphold fair labor standards, practice transparent and ethical operations, and can demonstrate low life-cycle environmental impacts (e.g.: reduced carbon footprint, minimal packaging, recyclability, safe end-of-life disposal). |
|  | **9.2**  The healthcare facility shall write clear sustainability requirements into every supplier contract, check regularly that the suppliers meet these requirements, require fixes, and replace suppliers that fail to improve and comply with the set requirements. |
| **Environmental Performance Monitoring and Continuous Improvement** | **10.1**  The healthcare facility shall track clearly defined environmental and climate-health indicators and review the data at least monthly to verify compliance with national standards and/or internal targets. |
|  | **10.2**  Planned internal audits and field inspections shall be carried out at defined intervals to confirm that environmental controls and emergency systems operate as intended. Findings shall be documented and reported without delay. |
|  | **10.3**  At least once a year, the healthcare facility’s top management shall review monitoring results, audit outcomes, and emerging risks to evaluate the continuing suitability, adequacy, and effectiveness of the facility’s environmental performance and approve a documented improvement plan for the next cycle. |
|  | **10.4**  All non-conformities identified through audits, incidents, or emergencies shall trigger tracked corrective actions. Lessons learned shall be captured and fed into the monthly reviews and the annual improvement plan. |
|  | **10.5**  All documents and records relating to environmental policies, plans, monitoring, and improvement actions shall be properly identified, approved, protected, and kept up to date. |

**Supplementary Table 2 - Legal Prioritization of Standards into Phases**

| **Theme** | **Phase 1 (Urgent & Mandatory)** | **Phase 2 (Later)** | **Key Legal Hooks in Lebanon** |
| --- | --- | --- | --- |
| 1. Environmental & Sustainability Policy | 1.2, 1.3 | 1.1, 1.4 | Law 444/2002 (Environmental Protection); Environmental Impact Assessment (EIA) / Strategic Environmental Assessment (SEA) readiness; Environmental Compliance for Establishments (ECE) Decree 8471/2012 |
| 2. Social Responsibility | 2.2 | 2.1, 2.3 | Law 444/2002 (Environmental Protection); Environmental Compliance for Establishments (ECE) Decree 8471/2012 |
| 3. Air Quality Management | 3.1, 3.2, 3.3 | 3.4, 3.5 | Ministry of Environment (MoE) Decision 52/1 (1996); MoE Decision 8/1 (2001); Law 78/2018 (Air Quality Protection) |
| 4. Water & Sanitation | 4.1, 4.2, 4.4 | 4.3, 4.5, 4.6 | Water Law 221/2000 (and amendments 241/2000, 337/2001); MoE Decision 52/1 (1996); MoE Decision 8/1 (2001) |
| 5. Waste Management | 5.1–5.4 | 5.5–5.6 | Decree-Law 13389/2004 (classification of medical waste); Law 80/2018 (Integrated Solid Waste Management); Law 64/1988 (Hazardous Waste Management) |
| 6. Energy Conservation & Efficiency | *Compliance with Emission Limit Values (ELVs) through Air Quality clauses 3.1–3.3* | 6.1–6.5 | National Energy Efficiency and Renewable Energy Action Plan (NEEAP); Revised Hospital Accreditation Standards (2022) |
| 7. Emergency Preparedness & Resource Planning | *If Environmental Impact Assessment (EIA) / Strategic Environmental Assessment (SEA) is triggered*: 7.1–7.4 | 7.5–7.6 | Decree 8633/2012 (EIA); Decree 8213/2012 (SEA); Revised Hospital Accreditation Standards (2022) |
| 8. Community Engagement | — (unless linked to EIA/SEA) | 8.1–8.4 | Decree 8633/2012 (EIA); Decree 8213/2012 (SEA); Revised Hospital Accreditation Standards (2022) |
| 9. Sustainable Procurement | *When hazardous or healthcare waste (HCW) services are procured* | 9.1–9.2 | Decree-Law 13389/2004 (medical waste); Law 80/2018 (Integrated Solid Waste Management) |
| 10. Environmental Performance Monitoring | 10.1–10.5 (*required for Environmental Compliance for Establishments proof*) | — | Decree 8471/2012 (ECE); Law 444/2002 (Environmental Protection) |
| **Compliance with Emission Limit Values (ELVs):** Legally binding under Ministry of Environment Decision 52/1 (1996) and Decision 8/1 (2001). Addressed in Air Quality Management clauses 3.1–3.3. Energy clauses reinforce this indirectly but are not the legal basis. | | | |
| **If Environmental Impact Assessment (EIA) / Strategic Environmental Assessment (SEA) is triggered:** Decree 8633/2012 (EIA) and Decree 8213/2012 (SEA) apply to new projects, expansions, or major upgrades. Emergency Preparedness clauses 7.1–7.4 become mandatory only in such cases. | | | |
| **When hazardous or healthcare waste (HCW) services are procured:** Procurement clauses 9.1–9.2 are mandatory when hospitals contract hazardous waste or healthcare waste services, which must comply with Decree-Law 13389/2004 and Law 80/2018. For other procurement categories, sustainability criteria are Phase 2 (non-mandatory). | | | |
| **Environmental Compliance for Establishments (ECE) proof:** Monitoring clauses 10.1–10.5 provide the documentation and audit trails required by Decree 8471/2012. These serve as legal proof of compliance for licensing. | | | |
